# Pre Versus Post Implementation of a Pharmacologic Antishivering Protocol During Targeted Temperature Management Following Cardiac Arrest

**DOI:** 10.1007/s12028-021-01327-9

**Published:** 2021-09-08

**Authors:** Calvin Huynh, Jevons Lui, Vala Behbahani, Ashley Thompson Quan, Amanda Morris, Laura Baumgartner

**Affiliations:** 1 Department of Pharmaceutical Services, University of California, San Francisco Medical Center, San Francisco, CA USA; 2 Sutter Eden Medical Center, Castro Valley, CA USA; 3 RWJBarnabas Health, Livingston, NJ USA; 4 Department of Clinical Sciences, Touro University California College of Pharmacy, Vallejo, CA USA

**Keywords:** Induced hypothermia, Shivering, Sudden cardiac death, Post cardiac arrest syndrome

## Abstract

**Background:**

Targeted temperature management (TTM) is endorsed by various guidelines to improve neurologic outcomes following cardiac arrest. Shivering, a consequence of hypothermia, can counteract the benefits of TTM. Despite its frequent occurrence, consensus guidelines provide minimal guidance on the management of shivering. The purpose of this study was to evaluate the impact of a pharmacologic antishivering protocol in patients undergoing TTM following cardiac arrest on the incidence of shivering.

**Methods:**

A retrospective observational cohort study at a large academic medical center of adult patients who underwent TTM targeting 33 °C following out-of-hospital (OHCA) or in-hospital cardiac arrest (IHCA) was conducted between January 2013 and January 2019. Patients were included in the preprotocol group if they received TTM prior to the initiation of a pharmacologic antishivering protocol in 2015. The primary outcome was incidence of shivering between pre- and postprotocol patients. Secondary outcomes included time from arrest (IHCA) or admission to the hospital (OHCA) to goal body temperature, total time spent at goal body temperature, and percentage of patients alive at discharge. All pharmacologic agents listed as part of the antishivering protocol were recorded.

**Results:**

Fifty-one patients were included in the preprotocol group, and 80 patients were included in the postprotocol group. There were no significant differences in baseline characteristics between the groups, including percentage of patients experiencing OHCA (75% vs. 63%, *p* = 0.15) and time from arrest to return of spontaneous circulation (17.5 vs. 17.9 min, *p* = 0.96). Incidence of patients with shivering was significantly reduced in the postprotocol group (57% vs. 39%, *p* = 0.03). Time from arrest (IHCA) or admission to the hospital (OHCA) to goal body temperature was similar in both groups (5.1 vs. 5.3 h, *p* = 0.57), in addition to total time spent at goal body temperature (17.7 vs. 18 h, *p* = 0.93). The percentage of patients alive at discharge was significantly improved in the postprotocol group (35% vs. 55%, *p* = 0.02). Patients in the postprotocol group received significantly more buspirone (4% vs. 73%, *p* < 0.01), meperidine (8% vs. 34%, *p* < 0.01), and acetaminophen (12% vs. 65%, *p* < 0.01) as part of the pharmacologic antishivering protocol. Use of neuromuscular blockade significantly decreased post protocol (19% vs. 6%, *p* = 0.02).

**Conclusions:**

In patients undergoing TTM following cardiac arrest, the implementation of a pharmacologic antishivering protocol reduced the incidence of shivering and the use neuromuscular blocking agents. Prospective data are needed to validate the results and further evaluate the safety and efficacy of an antishivering protocol on clinical outcomes.

## Introduction

Following cardiac arrest, cerebral ischemia occurs within 5 min of cessation of cerebral blood flow, leading to a cascade of deleterious reactions that result in neurologic injury. Secondary neurologic injury begins immediately after return of spontaneous circulation (ROSC) and persists for hours and days following cardiac arrest, further exacerbating neurologic insult [1, 2]. Targeted temperature management (TTM) has been shown in two randomized controlled trials to improve neurologic outcomes (with one trial demonstrating improved survival) in patients who remain in a coma following ROSC. TTM has been endorsed by the International Liaison Committee on Resuscitation, the American Heart Association, and the American Academy of Neurology as standard of care post cardiac arrest [3–7]. Current guidelines recommend controlling core body temperature between 32 and 36 °C [3–5].

Shivering is a major complication of TTM and is the body’s innate thermoregulatory response to decreases in core body temperature below a threshold of approximately 36 °C. Shivering can negate the neuroprotective benefits of TTM by increasing systemic and cerebral energy consumption, increasing metabolic demand, and making it difficult to achieve and maintain target temperature [1, 2, 8]. The incidence of shivering is reported to occur in up to 40% of patients undergoing TTM, yet despite the high incidence of shivering and negative consequences, consensus guidelines provide minimal guidance on the management of shivering [3–5, 9]. The Neurocritical Care Society guideline on the implementation of TTM provides general recommendations to monitor shivering using the Bedside Shivering Assessment Scale (BSAS) and to adopt a stepwise approach to shivering by prioritizing nonsedating interventions over opioid analgesics, sedatives, or paralytics, but it lacks specific pharmacologic protocols [8]. The Columbia Antishivering Protocol, published by Choi et al. [10], is an example of a stepwise pharmacologic algorithm that emphasizes use of the least sedating regimen to achieve adequate shiver control. This protocol consisted of scheduled acetaminophen, buspirone, and magnesium sulfate to replenish the serum magnesium level to a goal of 3–4 mg/dL and skin counterwarming prior to the initiation of cooling. Patients who demonstrated moderate-to-severe shivering on current interventions were escalated to dexmedetomidine or opioid for mild sedation, dexmedetomidine and opioid for moderate sedation, propofol for deep sedation, and neuromuscular blockade, sequentially [10].

No studies to date have evaluated the impact of a pharmacologic antishivering protocol in patients undergoing TTM post cardiac arrest. In 2015, our institution adopted a protocolized approach to shivering, incorporating nonpharmacologic and pharmacologic interventions that prioritized nonsedating medications. The purpose of this study was to evaluate the impact of a pharmacologic antishivering protocol on the incidence of shivering in critically ill patients post cardiac arrest during TTM.

## Methods

### Patients

This retrospective observational cohort study was conducted at the University of California, San Francisco (UCSF) Medical Center (San Francisco, CA, USA), which is an academic quaternary care hospital designated as a receiving hospital for acute myocardial infarctions that offers 24–7 cardiac catherization laboratory services. This study examined patients undergoing TTM following cardiac arrest before and after implementation of a pharmacologic antishivering protocol between January 2013 and January 2019. Patients were assigned to the preprotocol group if they received TTM between January 2013 and September 2015, prior to the implementation of a pharmacologic antishivering protocol. Patients who received TTM between September 2015 and January 2019 were assigned to the postprotocol group. Eligible patients included adults at least 18 years of age who experienced either out-of-hospital cardiac arrest (OHCA) or in-hospital cardiac arrest (IHCA), achieved ROSC, and were initiated on TTM. Patients were excluded if they died during the first 24 h following TTM initiation, were pregnant, received TTM for reasons other than post cardiac arrest, or had incomplete BSAS assessments documented in the electronic health record. This study was approved by the UCSF Human Research Protection Program Institutional Review Board. For this type of study, formal consent was not required.

### Data Collection

**Fig. 1:**
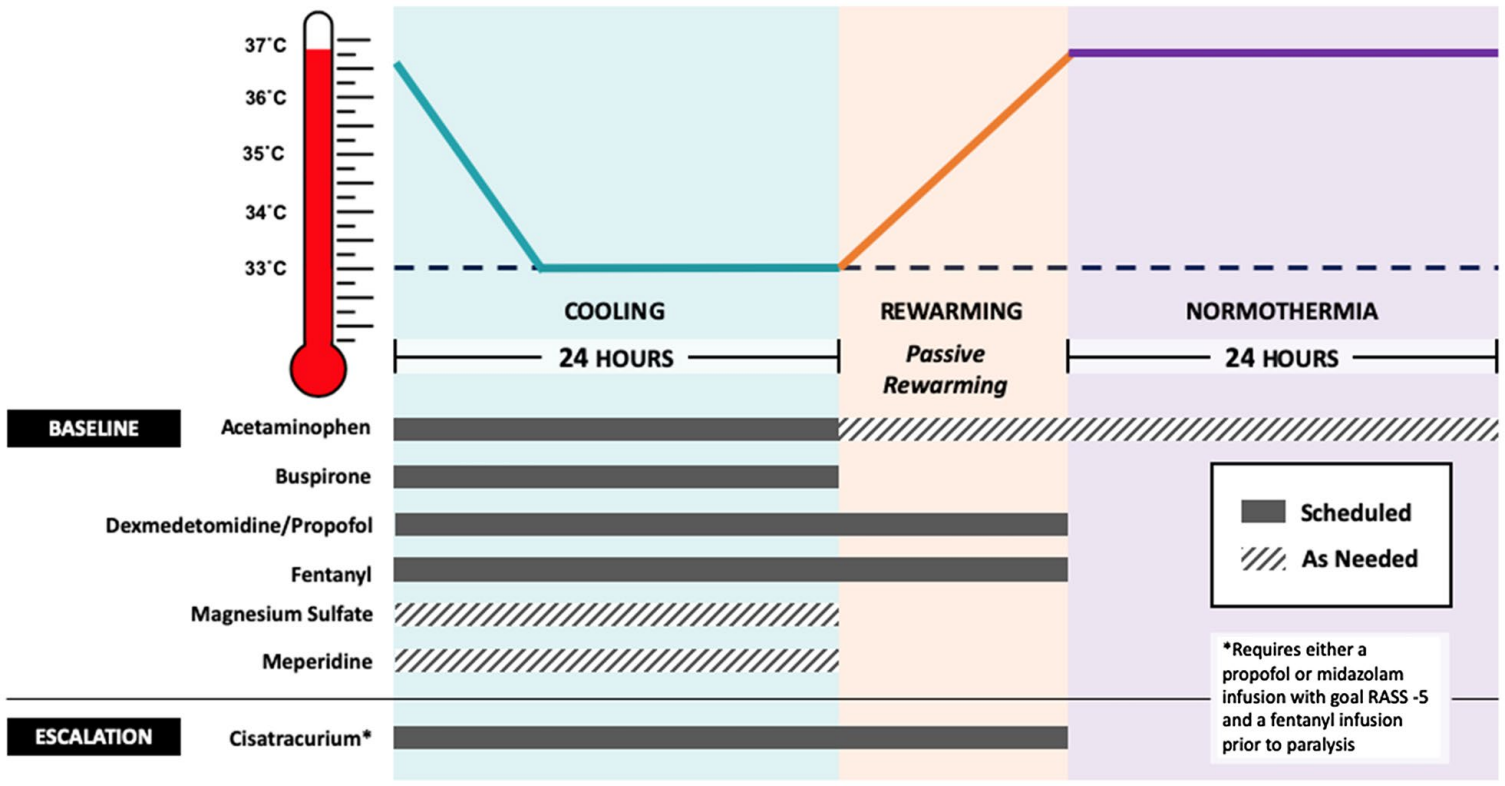
University of California, San Francisco Medical Center pharmacologic antishivering protocol by phases. The cooling phase includes induction, which was achieved as soon as possible, followed by maintenance at 33 °C. Rewarming was done passively by removal of cooling devices, and normothermia was maintained for at least 24 h following rewarming. RASS Richmond Agitation Sedation Scale

The electronic health record was used to identify patients who received TTM via the Arctic Sun Temperature Management System (Bard Medical, Covington, GA, USA) following cardiac arrest. Patient data were divided into three phases: cooling phase, rewarming phase, and normothermia phase (Fig. 1). The cooling phase began with the initiation of the Arctic Sun Temperature Management System and concluded at the start of rewarming. The rewarming phase began after the discontinuation of the Arctic Sun Temperature Management System and concluded when the patient reached a temperature of 36.5 °C. In the final phase, data were recorded for 24 additional hours after the patient was adequately rewarmed. Study data were collected and managed using Research Electronic Data Capture (REDCap) tools hosted at UCSF Medical Center [11]. REDCap is a secure Web-based platform designated to support data capture for research studies, providing (1) an intuitive interface for validated data entry, (2) audit trails for tracking data manipulation and export procedures, (3) automated export procedures for seamless data downloads to common statistical packages, and (4) procedures for importing data from external sources.

### TTM

Institutional protocols for the general management of TTM post cardiac arrest were unchanged before and after the implementation of a pharmacologic antishivering protocol for the duration of the study period. Patients were considered for TTM if they met inclusion criteria, which comprised age ≥ 18 years, cardiac arrest with ROSC and stable rhythm, not following commands after resuscitation, systolic blood pressure ≥ 90 mm Hg either spontaneously or with vasoactive medications, and known time of arrest. Pregnancy and known severe coagulopathy or active bleeding were considered to be relative exclusions to TTM, whereas other causes of coma and known terminal illness preceding the arrest were deemed strict exclusions to TTM.

Cooling was done to a target temperature of 33 °C and accomplished by use of the Arctic Sun Temperature Management System, in addition to ice packs applied to the trunk, axillae, and groin. Cold normal saline infusions were recommended if the goal temperature was not reached within 6 h but left to provider discretion. Temperature was preferentially monitored through placement of a temperature-sensing foley catheter. Patients were cooled for a total of 24 h from the initiation of cooling and allowed to passively rewarm to a goal temperature of 36.5 °C. Shivering was assessed using the BSAS, which was recorded every 30 min for the first 6 h and every hour until rewarming temperature was reached. Nurses were asked to measure the BSAS score by observing patients for a period of 2 min, during which they visually inspected and palpated the neck, thorax, arms, and legs. A score of 0 was assigned if no shivering was noted. A score of 1, or mild shivering, was assigned if shivering was localized to the neck or thorax only. A score of 2, or moderate shivering, was assigned if shivering involved gross movement of the upper extremities in addition to the neck and thorax. A score of 3, or severe shivering, was assigned if shivering involved gross movements of the trunk and upper and lower extremities [12]. Normothermia was advised for 24 h following rewarming.

### Antishivering Protocol

In September 2015, a pharmacologic antishivering protocol was introduced into the institutional TTM protocol (Table 1). Prior to its introduction, continuous infusion sedation, analgesia, and neuromuscular blocking agents (NMBAs) were highly advised prior to the initiation of cooling by institutional protocol but left at the discretion of the critical care providers. Under the preprotocol group, midazolam and propofol were considered first-line sedatives, whereas fentanyl was the recommended first-line analgesic. Similar to the Columbia Antishivering Protocol, the goal was to develop an antishivering protocol that incorporated the use of nonsedatives, such as acetaminophen, buspirone, meperidine, and magnesium, and to minimize the need for NMBAs. In addition, we transitioned to dexmedetomidine and propofol as our first-line sedatives because both have been shown to decrease the shivering threshold and, unlike midazolam, do not carry a high risk of delayed awakening following TTM [2, 13, 14]. We diverged from the Columbia Antishivering Protocol by recommending sedation, with either of the aforementioned agents, and analgesia with fentanyl at the onset of therapeutic hypothermia to prevent discomfort associated with TTM (Fig. 1) [13]. Skin counterwarming was employed both before and after implementation of the pharmacologic antishivering protocol. If shivering was not controlled with baseline interventions, an NMBA could be used, with cisatracurium being the preferred agent. Sedation with either propofol or midazolam, titrated to deep sedation, was required prior to neuromuscular blockade initiation.

**Table 1:** UCSF Medical Center pharmacologic antishivering protocol

	Intervention	Dose
Baseline	Scheduled	
	Acetaminophen	1000 mg per FT or IV Q6H^a^
	Buspirone	30 mg per FT Q8H
	Dexmedetomidine	0–1.2 μg/kg/h, targeting RASS^b^ score of − 3 (range − 4 to − 2)
	Propofol	0–75 μg/kg/min, targeting RASS^b^ score of − 3 (range − 4 to − 2)
	Fentanyl	25 μg IV Q15min PRN for pain and 0–100 μg/h titrated to CPOT^c^ < 3
	As needed	
	Magnesium sulfate	2 g IV Q6H PRN for serum magnesium < 2.5 mg/dL
	Meperidine	12.5–25 mg IV Q4H PRN for BSAS > 0 × 24 h (not to exceed 75 mg/24 h)
Escalation	Cisatracurium	0.15–0.2 mg/kg IV once followed by 0–5 μg/kg/min titrated to BSAS score of 0 (requires either a propofol or midazolam infusion with goal RASS score of − 5 and a fentanyl infusion prior to paralysis)

*CPOT* Critical Care Pain Observation Tool, *FT* feeding tube, *IV* intravenously, *PRN* as needed, Q15min, every 15 min,﻿ Q4H, every 4 h, Q6H, every 6 h, Q8H, every 8 h, *RASS* Richmond Agitation Sedation Scale

^a^500 mg per FT or IV Q6H for patients with hepatic insufficiency

^b^RASS scores: − 5, unarousable sedation; − 4, deep sedation; − 3, moderate sedation; − 2, light sedation; − 1, drowsy; 0, alert and calm; 1, restless; 2, agitated; 3, very agitated; 4, combative

^c^CPOT ≤ 2, minimal to no pain present; CPOT > 2, unacceptable level of pain

### Outcomes

The primary outcome of this study was to compare the incidence of shivering in patients before and after implementation of a pharmacologic antishivering protocol. Shivering was defined as a positive BSAS score (i.e., BSAS score > 0) at any time during the cooling phase. Secondary outcomes included induction time on the Arctic Sun Temperature Management System (defined as the time span between the initiation of the Arctic Sun Temperature Management System to the time goal temperature was reached), time from arrest (IHCA) or admission to the hospital (OHCA) to goal body temperature, total time spent at goal body temperature (32–34 °C), time to normothermia during rewarming, incidence of fever during rewarming, and disposition at discharge. All pharmacologic agents listed in the antishivering protocol were recorded. The receipt of each medication was confirmed in reviewing the medication administration record.

### Statistical Analysis

Continuous data are presented as mean ± standard deviation, and categorical data are presented as frequency and percentage, unless otherwise noted. Continuous variables were compared using a Student’s *t*-test or Mann–Whitney *U*-test, and categorical data were compared using a χ^2^ test or Fischer’s exact test, as appropriate. A sample size calculation was completed a priori to the initiation of the study. It was determined that we would need 82 patients (41 in each group) for 80% power, an alpha level of 0.05, and a 30% effect size to detect a difference in our primary end point. All statistical analysis was conducted using SAS 9.3 (SAS Institute, Inc, Cary, NC), and significance was set at *p* < 0.05.

## Results

Over the study period, a total of 131 patients who met inclusion and exclusion criteria were identified, with 51 patients in the preprotocol group and 80 patients in the postprotocol group. Patient demographics were similar between groups, including age (70 vs. 65.2 years, *p* = 0.09), sex (67% vs. 59% male, *p* = 0.36), and body mass index (27.9 vs. 26.4, *p* = 0.17). The majority of patients enrolled in this study experienced OHCA (75% vs. 63%, *p* = 0.15), with a mean time to ROSC of 17.5 vs. 17.9 min, respectively (*p* = 0.96). No difference was observed in the proportion of patients who experienced a shockable rhythm as opposed to a nonshockable rhythm as their initial rhythm (24% vs. 31% and 72% vs. 68%, respectively, *p* = 0.42). Length of stay in the intensive care unit was similar between groups, with a trend toward increased length of stay in the postprotocol group (7.3 vs. 9.3 days, *p* = 0.17). Three patients in the postprotocol group were deviated from protocol and had a target temperature of 36 °C while undergoing TTM as directed by the neurology consultation service, whereas all patients had a target temperature of 33 °C in the preprotocol group (*p* = 0.28) (Table 2).

**Table 2:** Demographic data

	Preprotocol Patients (*n* = 51)	Postprotocol Patients (*n* = 80)	*p* value
Age (years), mean ± SD	70 ± 18	65.2 ± 15.5	0.09
Male sex, *n* (%)	34 (67)	47 (59)	0.36
Body mass index, mean ± SD	27.9 ± 5.9	26.4 ± 6.8	0.17
Baseline serum magnesium (mg/dL), mean ± SD	2.1 ± 0.34	2.1 ± 0.4	0.89
Out-of-hospital cardiac arrest, *n* (%)	38 (75)	50 (63)	0.15
Initial rhythm, *n* (%)			
Pulseless electrical activity or asystole	37 (72)	54 (68)	0.42
Pulseless ventricular tachycardia or ventricular fibrillation	12 (24)	25 (31)	
Unknown	2 (4)	1 (1)	
Time to return of spontaneous circulation (minutes), mean ± SD	17.5 ± 12	17.9 ± 21.3	0.96
Targeted temperature, *n* (%)			
33 °C	51 (100)	77 (96%)	0.28
36 °C	0 (0)	3 (4%)	
ICU length of stay (days), mean ± SD	7.3 ± 8.3	9.3 ± 11	0.17

*ICU* intensive care unit, *SD* standard deviation

The number of patients who experienced shivering was significantly reduced in the postprotocol group compared with the preprotocol group (Table 3). Fifty-seven percent of patients in the preprotocol group had a positive BSAS score during the cooling phase, compared with 39% in the postprotocol group (*p* = 0.03). In addition, there was a significant reduction in patients with a maximal BSAS score of 2 (29% vs. 15%, *p* = 0.03) but no reduction in patients with a maximal BSAS score of 3 (6% vs. 8%, *p* = 0.35).

**Table 3:** Clinical outcomes between preprotocol vs. postprotocol patients undergoing TTM following cardiac arrest

	Preprotocol Patients (*n* = 51)	Postprotocol Patients (*n* = 80)	*p* value
*Cooling phase*			
Time to initiation of cooling (hours), mean ± SD	2.4 ± 1.8	2.7 ± 1.8	0.33
Time to goal temperature (hours), mean ± SD	5.1 ± 3.2	5.3 ± 3.9	0.57
Induction time on Arctic Sun (hours), mean ± SD	3 ± 2.4	3 ± 3.3	0.6
Total time at goal temperature (hours), mean ± SD	17.7 ± 5.7	18 ± 6.5	0.93
Total time on Arctic Sun (hours), mean ± SD	22 ± 4.3	23.2 ± 3.5	0.1
Serum magnesium (mg/dL), mean ± SD	2 ± 0.3	1.96 ± 0.4	0.2
Positive BSAS score (BSAS score > 0 at any time), *n* (%)	29 (57)	31 (39)	0.03
Maximum score = 1	11 (22)	13 (16)	0.5
Maximum score = 2	15 (29)	12 (15)	0.03
Maximum score = 3	3 (6)	6 (8)	0.35
Adjunct medication use, *n* (%)			
Acetaminophen	6 (12)	52 (65)	< 0.01
Buspirone	2 (4)	58 (73)	< 0.01
Meperidine	4 (8)	26 (34)	< 0.01
Total dose of adjunct medication received during cooling, median (IQR)			
Acetaminophen (mg)	1,750 (750–2,750)	3,000 (1,875–3,000)	0.12
Buspirone (mg)	45 (37.5–52.5)	60 (60–90)	0.34
Meperidine (mg)	37.5 (25–50)	25 (12.5–25)	0.31
Sedative and analgesic use, *n* (%)			
Dexmedetomidine	2 (4)	15 (19)	0.02
Fentanyl	43 (84)	64 (80)	0.65
Midazolam	7 (14)	6 (8)	0.4
Propofol	49 (96)	63 (79)	0.01
Total doses of sedatives and analgesics received during cooling, median (IQR)			
Dexmedetomidine (μg/kg)	5 (4–6)	3.7 (3.3–7.2)	0.45
Fentanyl (μg/kg)	12.1 (3.4–19.5)	13.8 (8.5–24.5)	0.08
Midazolam (mg)	5 (2–11)	10.5 (2–22.5)	0.42
Propofol (mg/kg)	56.5 (20.2–103)	54 (36–72)	0.14
Use of a continuous infusion neuromuscular blocking agent, *n* (%)	10 (19)	5 (6)	0.02
*Rewarming phase*			
Time to rewarming goal temperature (hours), mean ± SD	14.7 ± 9	16.6 ± 12.2	0.37
Incidence of fever during rewarming (> 37.5 °C), n (%)	20 (39)	20 (25)	0.06
*Clinical outcomes*			
Alive at discharge, *n* (%)	19 (35)	44 (55)	0.02

*BSAS* Bedside Shivering Assessment Scale, *IQR*, interquartile range, *SD*, standard deviation

During the cooling phase, induction time on the Artic Sun Temperature Management System was similar between the two groups at 3 h (*p* = 0.6), as was time from arrest (IHCA) or admission (OHCA) to goal temperature (5.1 vs. 5.3 h, *p* = 0.57). Total time on the Artic Sun Temperature Management System also did not differ between the two groups (22 vs. 23.2 h, *p* = 0.1), nor did time at goal temperature (17.7 vs. 18 h, *p* = 0.93).

During rewarming, time from initiation of rewarming to normothermia was similar between groups (14.7 vs. 16.6 h, *p* = 0.37). Fevers occurred in 39% of patients in the preprotocol group and 25% of patients in the postprotocol group, which was not statistically significant (*p* = 0.06).

Acetaminophen, buspirone, and meperidine were most frequently used in the postprotocol group compared with the preprotocol group (*p* < 0.01). In the preprotocol group, the percentages of patients who received acetaminophen, buspirone, and meperidine were 12%, 4%, and 8%, respectively (Table 3). Use of each agent increased in the postprotocol group to 65%, 73%, and 34%, respectively. No difference was observed in the use of fentanyl (84% vs. 80%, *p* = 0.65) and midazolam infusions (14% vs. 8%, *p* = 0.4) between groups. However, a statistically significant increase in the use of dexmedetomidine (4% vs. 19%, *p* = 0.02) and a statistically significant decrease in the use of propofol (96% vs. 79%, *p* = 0.01) was observed in the postprotocol group. No difference was observed in the total doses of sedatives and analgesics used during cooling between groups. Use of NMBAs significantly declined in the postprotocol group (19% vs. 6%, *p* = 0.02). More patients were alive at discharge in the postprotocol group as compared with the preprotocol group (35% vs. 55%, *p* = 0.02).

## Discussion

Minimizing shivering during TTM is important to reduce secondary brain injury and to maintain efficacy of TTM following cardiac arrest [1, 8]. Although guidelines recognize the importance of adequately controlling shivering, they provide minimal guidance on a concrete approach, stemming from a paucity of data. The findings of this study indicate that a protocolized approach to pharmacotherapy interventions for patients undergoing TTM post cardiac arrest can reduce shivering in addition to the need for NMBAs.

Baseline interventions were selected based on evidence supporting their ability to lower the shivering threshold as well as their favorable safety profile. Acetaminophen is an antipyretic that is believed to act on the hypothalamic heat-regulating center and has been shown to decrease core body temperature and reduce shivering [15–17]. Buspirone is a 5-HT agonist that is thought to act on 5-HT_1A_ receptor to lower the shivering threshold. It has been shown to lower the shivering threshold as monotherapy, additively with dexmedetomidine, and synergistically with meperidine in healthy volunteers [18, 19]. Magnesium is an NMDA antagonist that has only modest reductions in the shivering threshold [20]. However, it has been demonstrated to increase the rate of cooling and improve patient comfort when using a surface cooling technique by reducing smooth muscle tone and thereby counteracting the normal adaptive response to surface cooling of vasoconstriction [2, 21]. Meperidine, like other opioids, has been demonstrated to lower the shivering threshold but differs in that it has additional antishivering action at equianalgesic doses and inhibits shivering twice as much as vasoconstriction [2, 22]. In addition, it has been demonstrated to work additively or synergistically with other pharmacologic antishivering agents and additively with skin surface warming [23]. Following the introduction of our pharmacologic antishivering protocol, we observed an increase in the use of acetaminophen, buspirone, and meperidine in the postprotocol group, which corresponded to a decrease in the incidence of shivering.

Sedation and analgesia are considered baseline interventions important to prevent and treat discomfort associated with TTM [13]. In addition, Choi et al. [10] found that only 25% of patients undergoing TTM targeting hypothermia had shivering controlled with acetaminophen, buspirone, magnesium, and counterwarming alone, whereas 82% had shivering controlled with the addition of dexmedetomidine and an opioid. Dexmedetomidine and propofol were considered first-line sedatives and fentanyl as a first-line analgesic in the postprotocol group. Both dexmedetomidine and propofol have been demonstrated to lower the shivering threshold individually [24, 25]. Use of NMBAs was considered last line in the protocol because of considerable side effects associated with their use, including loss of neurologic examination and critical illness polyneuropathy [10]. Following the introduction of our pharmacologic antishivering protocol, we observed an expected increase in the use of dexmedetomidine and a decrease in the use of propofol. Total doses of sedatives and analgesics administered during cooling did not differ between groups, suggesting that the observed difference in the incidence of shivering is not due to higher doses of sedatives and analgesics. Use of NMBAs also declined in the postprotocol group.

There are several limitations to note, as this was a prepost retrospective observational study. First, management of patients undergoing TTM was at the complete discretion of the provider. Providers had the ability to omit certain elements of the pharmacologic antishivering protocol or initiate NMBA therapy from the onset of TTM. This is best illustrated in the preprotocol group, in which an unexpectedly low percentage of patients received NMBAs as recommended by our institutional protocol. However, use of the individual pharmacologic antishivering agents in the postprotocol group was notably higher, except for use of NMBAs, suggesting greater protocol adherence. Second, the preprotocol group included patients from 2013 to 2015, whereas the postprotocol group included patients from 2015 to 2019. This prepost design makes our study susceptible to the influence of overall changes in practice or attitudes toward TTM over the years that may have influenced the outcomes seen in this study. This includes greater awareness of the negative impacts of shivering and the necessity to act on positive BSAS scores. Third, we did not characterize or quantify adverse effects from the agents used and cannot comment on tolerability of our pharmacologic antishivering protocol. This is an important consideration, as meperidine and buspirone can both lower the seizure threshold in a population already prone to seizures. Fourth, we were unable to characterize the etiology of cardiac arrest, which may have influenced the observed improvement in mortality. However, there was no difference observed in shockable and nonshockable rhythms or in time to ROSC. Fifth, three patients in the postprotocol group had a target temperature of 36 °C during TTM. This deviation from the recommended 33 °C from our institutional protocol was done at the discretion of the neurology consultation service for reasons not explicitly captured through chart review. We believe the switch to 36 °C in these three patients would be unlikely to lead to a difference in shivering, as observed by Nielsen et al. [26]. Lastly, BSAS scores relied on nursing documentation, and in some instances, patients had to be excluded because of lack of complete BSAS documentation.

## Conclusions

In summary, this study demonstrates that the implementation of a pharmacologic antishivering protocol that focuses on minimizing use of NMBAs is feasible and leads to a decrease in the incidence of patients with shivering in those undergoing TTM following cardiac arrest. In addition, patients managed with the antishivering protocol may have an improvement in mortality. Additional larger prospective studies are needed to validate the findings of this study and evaluate the safety of these interventions and their impact on clinical outcomes.
